# Book Review

**Published:** 1931

**Authors:** 


### Reviews of Books

---

**Conduct of Life Assurance Examinations.** By E. M. Brockbank, M.D., F.R.C.P. Pp. viii., 172. London: H. K. Lewis & Co. Ltd. 1931. Price 7s. 6d.—To all who are engaged in the practice of examination for life assurance this book will prove a treasury of information. The details of the physical examination of the patient and the tests for albumen and sugar in the urine will be useful to the young practitioner, whilst all who take an intelligent interest in insurance work will read with benefit the author's remarks on impaired lives, overweight, hereditary disease, venereal disease, glycosuria and the various forms of albuminuria. The author's wide experience of insurance work enables him to give many valuable hints to the general practitioner, and to explain many small points in insurance procedure which are not always clear to the medical examiner. For the estimation of blood pressure Dr. Brockbank is an advocate of the mercurial sphygmomanometer, the auscultatory method, and recording the diastolic pressure at the point of complete silence. His remarks under "Radical Mastoid Operation" are obviously intended to apply to simple mastoidectomy, not the radical operation.

**The Medical Annual, 1931.** By Carey F. Coombs, M.D., F.R.C.P., and A. Rendle Short, M.D., B.S. Pp. xcix., 604. Illustrated. Bristol: John Wright & Sons Ltd. 1931. Price 20s.—The great importance of *The Medical Annual* to the practitioner does not need emphasis, for it provides the only way by which he can discover the useful new or improved methods of diagnosis and treatment which are scattered over the current literature of the medical world. To help the busy man still more the introductory chapter gives a summary of the most important advances of the year, and a marvellous index extends to nearly thirty pages of small type. Besides this there are lists of new books and appliances and the addresses of all kinds of institutions, homes, and medical societies. The present volume has a new departure, a section on diseases of the teeth, which will be welcomed by many readers. Among fresh methods of treatment we notice dehydration in severe epilepsy, the use of lactic acid in spring conjunctivitis, and of stramonium in post-encephalitic troubles. We find useful warnings about the spread of glandular and some forms of undulant fever, and botulismus. Great advances, too, are being made in the leukæmic diseases and in those connected with calcium metabolism. The ultimate results of gastro-enterostomy in 2,600 cases gives much food for thought and the abstract of the subject will interest most readers. Many of us know little of the powers of local authorities in the registration of nursing homes and as to the records which they can insist on being kept. This matter, and the necessity of medical practitioners registering their houses if they take in invalid patients, are discussed in a very useful article. The beautiful plates and illustrations in this volume are very numerous and most helpful.

**The History of Medicine.** By BERNARD DAWSON, M.D., F.R.C.S. Pp. xiv., 160. 31 illustrations. London: H. K. Lewis & Co. Ltd. 1931. Price 7s. 6d. This little volume comprises a course of seven lectures given to junior medical students in the University of Adelaide, N.S.W. Attendance on them was voluntary, but they were so popular that "all the students attended all the time." The author has given a very brief but good account of the gradual growth of medicine and surgery from prehistoric times to the present day, showing how it has been affected by the ups and downs of political history and by the lives of the great leaders of thought. The short sketches of the lives or work of these latter are admirable—Galen, Hippocrates and Avicenna are as interesting as Vesalius, Harvey and Hunter. A good deal of space had to be given up to explain the social and political state of each period, and on the whole this has been successful and clearly expressed, though we may not always agree with his view of history. We should have liked some reference to the debt the Arabist medicine owed to Indian Buddhism and to the influence of the school of Gondishapor, and again the revival of hospitals in the eighteenth century, and the growth of medical schools and societies should not be forgotten; but the author has done well to limit his subjects, and he has succeeded in writing a most interesting story.

**Pye's Surgical Handicraft.** By H. W. Carson, F.R.C.S. Tenth Edition. Pp. xviii., 641. 343 illustrations. Bristol: John Wright & Sons Ltd. 1931. Price 21s.—We welcome the appearance of another edition of this well-known work. It is now seven years since the last revision took place, yet without sacrificing the individuality of its original author, this surgical classic has once more been brought into line with modern progress. The Editor, Mr. Herbert Carson, whose untimely death took place before this volume left the press, has been meticulously careful in his work of revision, and he has been assisted by a number of distinguished specialists who have reviewed their particular subject. One small omission to which we would call attention is the absence of any mention of the use of the de Pezzer catheter in the treatment of acute urinary retention. We have nothing but praise for this work, which should find its place on the bookshelves, not only of house surgeons, for whom it was primarily intended, but of every general practitioner.

**Radium Therapy: Principles and Practice.** By G. E. Birkett, M.C., M.R.C.S. Pp. x., 186. 52 illustrations. London: Cassell & Co. Ltd. 1931. Price 17s. 6d.—A special interest attaches to this book as it represents the ripe experience of one who had devoted the latter years of his life to the study of this subject, and who died suddenly and unexpectedly when this work had been completed. The book is eminently practical and clinical, and therefore the theoretical physics underlying the action of radium is reduced to a minimum. The general attitude of the author towards the subject is one of a very sane and moderate optimism. We must be content for the present to treat and alleviate the sufferer from cancer, but we cannot yet claim to cure the disease. The methods of protection are rightly given in some detail. The possibility of burns of the skin or of necrosis of the bone coming on at long periods after exposure is interesting and rather depressing. In cancer of the mouth radium gives just as good or better results than excision, with much less mutilation. The problem of the glands is, however, very difficult. It is here suggested that a block dissection should be done and radium needles implanted in the wound in all cases when glands are palpably affected. It is not considered that such treatment will cause any difficulty in wound healing. In cases where there is no manifest affection of the glands an external application is sufficient. In regard to the breast we are glad to note that it is stated that there is no justification for withholding operation from early and favourable cases. The opinion in regard to the value of radium in cancer of the rectum is a very guarded one, and it is only in regard to epitheliomata, cancer of the cervix and certain sarcomata that the author is really enthusiastic. He holds that there is no justification for the practice of routine biopsy, because this operation has a bad effect on the growth, whilst it greatly increases the liability to dissemination. The book is a model of clear teaching and is full of excellent figures.

**Among the Thinkers.** By J. A. LINDSAY, M.D., F.R.C.P. Pp. ix., 197. London: H. K. Lewis & Co. Ltd. 1931. Price 5s.—Dr. Lindsay follows Lord Morley's cue: "A book which is worth reading is worth taking notes from." Writers draw alike from the cisterns of study and the springs of genius. Into that discursive book *The Doctor* Southey threw his collectanea. Sir Thomas Browne can be found in the rough in his commonplace books. If we have fagged and forgotten good things spoken, written or copied, yet, one who sold his library and kept his billiards we counted already dead to the depth and grandeur of life. This volume is such an anthology as a man of experience might leave to his friends. Here are the old oracles with the savour of their classic speech: latest exponents, too, of the themes of human faculty, character and suffering. We envy the author his first glow in transcribing many of these passages and aphorisms—acute and various as the conversation of Solomon's table.

**Primary Syphilis in the Female.** By THOMAS ANWYL DAVIES, M.D. Pp. x., 111. Illustrated. London: Oxford University Press. 1931. Price 12s. 6d.—This is a comprehensive and sound survey of a subject which has been inadequately treated so far in the literature. There are so many features of outstanding merit that it is difficult to select anything for special mention unless perhaps the excellent plates and illustrations. The author does not hesitate to break away from some of the historic ideas as to the commonest sites for genital sores. His dogmatic reference to the cervix is most refreshing in this connection, where his large experience must be unique. This book will be welcomed by all medical men as a solid contribution to this difficult subject. The amount of work devoted to the book, even allowing for the author's generous acknowledgment of help from others, must have been enormous, and the result is most successful.

